# Analysis of antibiotic-induced drug resistance of *Salmonella* enteritidis and its biofilm formation mechanism

**DOI:** 10.1080/21655979.2021.1988251

**Published:** 2021-12-02

**Authors:** Weiping Dai, Yuliang Zhang, Jianfeng Zhang, Chengyu Xue, Jun Yan, Xin Li, Xiaohua Zheng, Rui Dong, Jing Bai, Yi Su, Pinghui Xie, Weiming Zhong, Hongyang Zhang, Zibo Yan, Weiqi Zhong, Yu Song

**Affiliations:** a Institute for Disease Control and Prevention, Heilongjiang Provincial Center for Disease Control and Prevention, Harbin, Heilongjiang Province, China; b Department of Infectious Diseases, Infectious Disease Hospital of Heilongjiang Province, Harbin, Heilongjiang Province, China; c Food Science and Technology Branch, Heilongjiang Vocational College of Biology Science and Technology, Harbin, Heilongjiang Province, China

**Keywords:** *Salmonella enteritidis*, drug resistance, biofilm, fluorescence quantitative PCR (FQ PCR), drug resistance gene

## Abstract

This research was to explore antibiotic-induced drug resistance of *Salmonella enteritidis* and its biofilm formation mechanism. Kirby-Bauer (K-B) disk method recommended by Clinical and Laboratory Standards Institute (CLSI) was used to test drug sensitivity of Salmonella enteritidis to 16 kinds of antibiotics including ß-lactams, aminoglycosides, quinolones, sulfonamides, chloramphenicols, and tetracyclines. Polymerase chain reaction (PCR) was performed to detect carrying of drug resistance genes of 29 kinds of antibiotics including ß-lactams, aminoglycosides, quinolones, sulfonamides, chloramphenicols, and tetracyclines of *Salmonella enteritidis*. The expressions of esp, ebpA, ge1E, and fsrB genes in biofilm group and plankton group were detected when Salmonella was induced, and difference of gene expression was detected by FQ-PCR. The drug resistance rates of *Salmonella enteritidis* to nalidixic acid, ampicillin, streptomyces, and cefoperazone were high, which were 94.5%, 75%, 67%, and 52%, respectively. 94 strains of *Salmonella enteritidis* formed 22 kinds of drug resistance spectrum, the strains were generally resistant to 4-5 antibiotics, and some strains formed fixed drug resistance spectrum as follows: AMP-CFP-STR-NA-TE (22.6,21.7%), AMP-STR-NA-TE (17,16%), and AMP-CFP-STR-NA (11.1,10.6%). During biofilm formation, fsr can increase expression of ge1E and decrease expression of esp and ebpA. Consequently, *Salmonella enteritidis* was generally resistant to nalidixic acid, ampicillin, and streptomycin, and the multidrug resistance was severe. The drug resistance genes sul2, sul3, blaTEM-1-like, tet(A), and tet(G) were highly carried in *Salmonella enteritidis*. Esp, ebpA, ge1E, and fsrB genes were closely related to biofilm formation of *Salmonella enteritidis*.

## Graphical abstract

**Figure:**
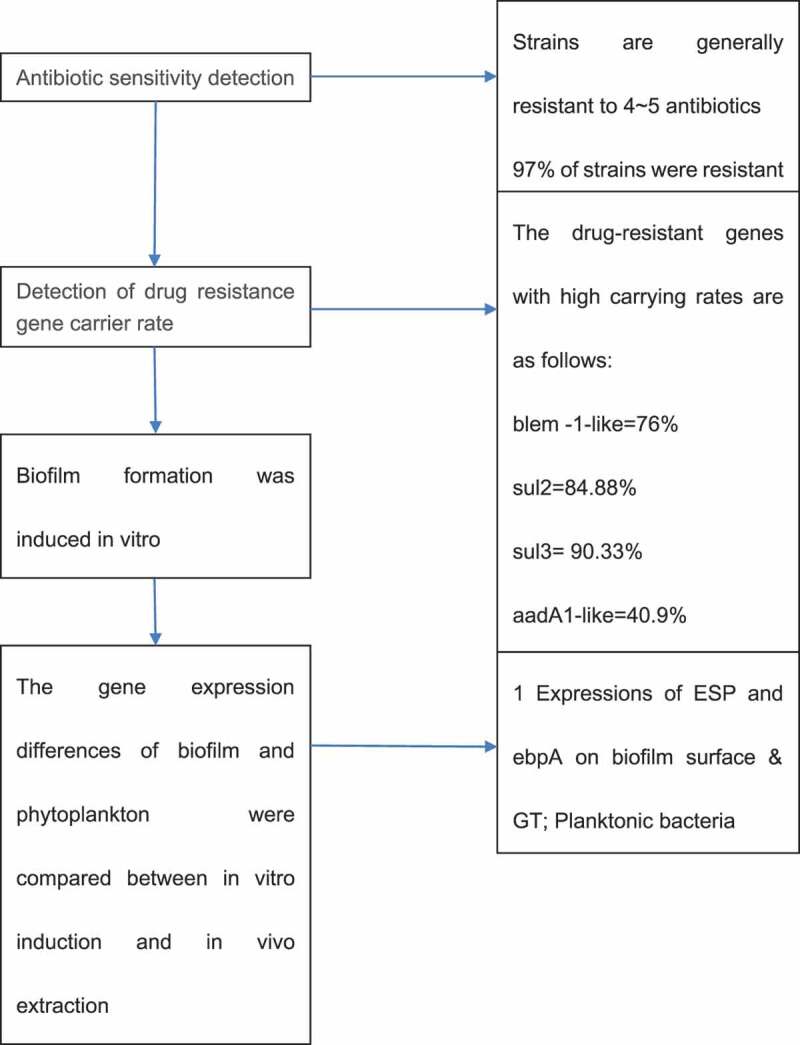

## 1. Introduction

*Salmonella* is a kind of enterobacteriaceae that parasitizes in the intestinal tract, which destroys intestinal epithelial cells to enter the blood circulation, causes systemic infection, and shows septicemia, bacteremia, diarrhea, and fever. It is widely distributed in nature with many different kinds, which can not only infect livestock and other animals, but also lead to acute, chronic, or recessive infectious diseases [1]. Moreover, it can also lead to human poisoning through contaminated meat, eggs, milk, and other animal-derived food [2]. China is a big country of animal husbandry. *Salmonella* will not only affect the economy and export of animal husbandry, but also threaten human health. At present, the prevention and treatment of *Salmonella* mainly depend on antibiotics. However, due to the extensive use of antibiotics in clinical, animal husbandry and veterinary, and animal-derived food processing, the drug resistance of *Salmonella* is increasing, and the phenomenon of multi-drug resistance (MDR) is also becoming more and more serious. *Salmonella* has an important impact on human society, so the monitoring of its drug resistance data is of great significance for analyzing the evolution of its drug resistance spectrum, guiding clinical medication, and preventing the infection and prevalence of *Salmonella* [3]. *Salmonella typhimurium* and *Salmonella* enteritidis are the most common *Salmonella enteritidis* that can infect humans.

The biofilm is the membrane-like complex composed of bacteria encapsulated by extracellular macromolecules and its secreted hydrated matrix formed during the growth of bacteria that adhere to the surface of living or inanimate objects. Research on *Salmonella* biofilm showed that almost all strains can form biofilm, and most *Salmonella* strains have strong ability to form biofilm [4]. Once biofilm is formed, the resistance of bacteria to adverse conditions such as drying, extreme temperature, antibacterial agents, and disinfectants is greatly enhanced, which leads to the long-term existence of *Salmonella* in the animal growth environment and the contamination of meat, eggs, and milk of animal husbandry [5]. Therefore, the study of biofilm formation mechanism is of great significance to prevent the spread and infection of *Salmonella*. At present, studies showed that the process of biofilm formation is divided into four stages: adhesion, growth, maturation, and release. Adhesion includes the adhesion of planktonic bacteria on the surface of medium and the aggregation of bacteria. The adhesion of bacteria generally consists of selective and nonselective forms. Generally, the adhesion mode on the surface of inanimate host is non selective, while that on the surface of living host is selective [6]. Nonselective adhesion is mainly mediated by adhesion factors and bacteria surface appendages, while selective adhesion refers to the specific recognition of host surface receptors by specific adhesion proteins on bacteria surface, so as to adsorb specific types of cells. Biofilm growth is a process in which bacteria attach to the surface of media and begin to form large diffusive structures [7]. The main regulatory role is played by extracellular polymeric substance (EPS) in the middle of the process. When the colony was expanded to a certain stage, it entered the mature stage of biofilm [8]. There are many research views on this stage, among which the widely accepted view is quorum sensing system (a signaling mechanism of bacteria regulating the expression of multi-target genes by detecting population cells density, thereby ensuring the transport of nutrients and the removal of waste in biofilms to avoid the lack of space and nutrients caused by excessive reproduction and growth of bacteria) [9]. Mature biofilm can be partially detached under the action of internal regulation mechanism or external scour force. The falling bacteria can further transform into planktonic growth state and form new biofilm after adhering to the suitable medium surface, which is the release of biofilm [10]. In summary, gene regulation has a great influence on the formation of biofilm.

Therefore, this paper takes *Salmonella enteritidis* as the research object to study its drug resistance and its drug resistance genes. Moreover, the formation of the biofilm of *Salmonella enteritidis* was simulated in vitro in this research, and the gene expression of the biofilm group and planktonic bacteria group were compared before and after the formation of *Salmonella enteritidis*. It was expected to provide reference and basis for better understanding the mechanism of biofilm formation. In addition, the drug-resistant genes of *Salmonella enteritis* were studied in order to obtain more information for the clinical application of antibiotics in the treatment of *Salmonella* infection.

## 2. Research materials and methods

### 2.1. Source of strains

A total of 94 strains of *Salmonella enteritidis* with different sources were involved in this research. A total of 32 strains came from the special monitoring of baseline survey of chicken and pig industry chain by the disease control system of Shaanxi province in recent 3 years. Among them, 2 strains were isolated from food of food poisoning, 7 strains were isolated from health carriers of food producers, and 17 strains were isolated from food-borne diarrhea. The strain was preserved by conventional liquid paraffin preservation method. All strains were identified by full-automatic microbial identification system VITEK that was produced by bioMerieux Inc (Hazelwood, Mo, USA). The quality control strain of drug sensitivity test was *Escherichia coli*, which was provided by Center for Disease Control and Prevention of Xi’an city.

### 2.2. Main reagents and equipment

Main reagents: Mueller Hinton (MH) agar, common nutrient agar, drug sensitivity test paper, sterile saline, and *Salmonella* diagnostic serum.

Main instruments and equipment: autoclave, 37°C constant temperature incubator, turbidimetric instrument, drug sensitive paper distributor, sterile cotton swab, tweezers, inoculation ring, test tube, slide, vernier caliper, etc.

### 2.3. Antibiotic drug sensitivity test

Antibiotic sensitivity test was carried out by Kirby-Bauer disk diffusion method [11] on the following antibiotics: penicillins: ampicillin (AMP, 10 μg), amoxicillin (AMC, 30 μg). Generation II cephalosporins: cefoxitin (FOX, 30 μg). Generation III cephalosporins: cefotaxime (CTX, 30 μg), cefoperazone (CFP, 35 μg), ceftriaxone (CRO, 30 μg). Generation IV cephalosporins: cefepime (FEP, 30 μg). Aminoglycosides: streptomycin (STR, 10 μg), gentamicin (GN, 10 μg), kanamycin (K, 30 μg), amikacin (AK, 30 μg). Quinolones, fluoroquinolones: nalidixic acid (NA, 30 μg), ciprofloxacin (CIP, 5 μg). Sulfonamides: sulfamethoxazole (SXT, 25 μg). Chloramphenicols: chloramphenicol (C,30 μg). Tetracyclines: tetracycline (TE, 30 μg), with a total of 16 species.

### 2.4. PCR amplification of drug resistance genes and results

DNA extraction: The method of bacterial DNA extraction was based on the DNA extraction kit (Dalian TAKARA Company, BK-ML-F96). The brief description was as follows: 1.5 mL bacterial culture medium was placed in 1.5 mL microcentrifuge tube, centrifuged at 12,000 rpm for 2 min, and the supernatant was poured out. 180 μL Buffer GL, 209 μL Proteinase K, and 10 μL RNase A were added successively, and then the metal bath was given at 56°C for 10 min. 200 μL Buffer GB and 200 μL 100% ethanol were added. The Spin Column was placed on the Collection Tube, the solution was moved to the Spin Column, centrifuged at 12,000 rpm for 2 min, and the filtrate was poured out. 500pL Buffer WA was added, the solution was centrifuged at 12,000 rpm for 1 min, and the filtrate was poured out. 700 μL Buffer WB was added, the solution was centrifuged at 12,000 rpm for 1 min, the filtrate was poured out, and the previous operation was repeated. The Spin Column was placed on the Collection Tube and the solution was centrifuged at 12,000 rpm for 2 min. The Spin Column was placed on a new 1.5 mL centrifuge tube and added with 50–200 μL sterilized water or Elution Buffer. The Spin Column was standing at room temperature for 5 min and centrifugated at 12,000 rpm for 2 min to elute DNA. The extracted DNA template was stored at −20°C as standby.

PCR amplification [12]: the PCR amplification system for drug resistance genes detection was shown in Table 1. PCR cycle parameters: pre-deformation at 95°C for 5 min, deformation for 1 min, annealing temperature and time were determined according to different primer, extension at 72°C for 1 min, extension at 72°C for 10 min after 30 cycles.

**Table 1.** PCR amplification reaction system for drug resistance genes

Reagent	Adding amount (μL)
10× PCR buffer	3
DdH_2_O	19
dNTP (2.5 mmol/L)	0.6
F (10 μmol/L)	1
R (10 μmol/L)	1
Taq DNA enzyme (1 U/μL)	0.3
Template	1
Total quantity	25

The PCR products of drug-resistant genes were detected by agarose gel electrophoresis with 1% concentration, stained with EB, and photographed with gel imager.

### 2.5. Laboratory induced biofilm formation

1. The sterile catheter was cut into a section of 2 cm, and the bacterial solution was cultured to the concentration of 0.5 McFarland in tryptic soy broth culture tube. After that, the catheter was placed in the tube and cultured overnight in a water bath at 37°C, and the tube was vibrated. 2. The catheter was taken out and slowly rinsed with sterile saline to remove the planktonic bacteria. Then, the rinsed catheter was put into the new tryptic soy broth culture tube and incubated overnight the same as the above steps. 3. The catheter was cultured using the above method for 7 days. 4. After the catheter was removed and rinsed, it was put into 3 mL sterile saline tube and the film was the shaken down by vortex vibrator. 5. The catheter was inoculated on Congo red culturing medium and whether biofilm was formed was identified [13].

### 2.6. Real-time fluorescent quantitative PCR reaction

1. Preparation of PCR reaction solution [14]: 12.5 μL SYBR Premix ExTaq, 0.5 μL PCR Forward Primer, 0.5 μL PCR Reverse Primer, 1 μL 16S-DNA primer, 0.5 μL Passive Reference DyeI/PCR Enhancer, 0.5 μL MgCl, Ccna, MgCl 0.5 μL, and cDNA template added with 1 μL sterilized double distilled water to make up to 25 μL were added to the marked reaction tube. The PCR reaction condition was pre-denaturation at 50°C for 2 min, denaturation at 95°C for 10 min, annealing at 95°C for 40 s, extension at 72°C for 40 s, with 45 cycles. The melting curve reaction conditions were as follows: 95°C 0 s, 20°C/s, 65°C 15 s, 20°C/s, 95°C oscillation, and 0.1°C/s. 2. The positive expression strains of four genes in the two groups of bacteria were selected from the reverse transcription results, and the RT-FQ-PCR was carried out according to the above method. 3. According to the above steps, fluorescent quantitative PCR was performed on the strains that had been induced forming biofilm and those had not been induced, and the differences in gene expression between them were analyzed. 4. *Salmonella enteritidis* biofilm induced in vitro and biofilm extracted in vivo were analyzed and tested by FQ PCR and the differences were analyzed in terms of genes expression.

## 3. Results

This study takes *Salmonella enteritidis* as the research object. Kirby-Bauer disk diffusion method was used to detect the resistance of *Salmonella enteritidis* from different sources to different antibiotics and the carrying rate of resistant genes. The results showed that 97% of the strains were resistant to at least one antibiotic. *Salmonella enteritidis* had the highest resistance rate to nalidixic acid, followed by ampicillin, streptomycin, and cefoperazone. Some strains mainly formed a fixed resistance spectrum: AMP-CFP-STR-NA-te (22.6,21.7%), AMP-STR-NA-TE (17,16%), AMP-CFP-STR-NA (11.1,10.6%). In addition, the biofilm of *Salmonella enteritidis* was formed by inducing in vitro, and its gene expression was compared with the biofilm extracted in vivo and planktonic bacteria. The results showed that the expression levels of esp and ebpA in biofilm group were 299 and 60 times higher than those in planktonic bacteria group, respectively. The expression of esp, ebpA, gl1E, and fsrB before and after biofilm formation in vitro were significantly lower than those formed in vivo.

### 3.1. *Drug sensitivity results of* Salmonella *enteritidis strain K-B disk method*

**Figure 1.**
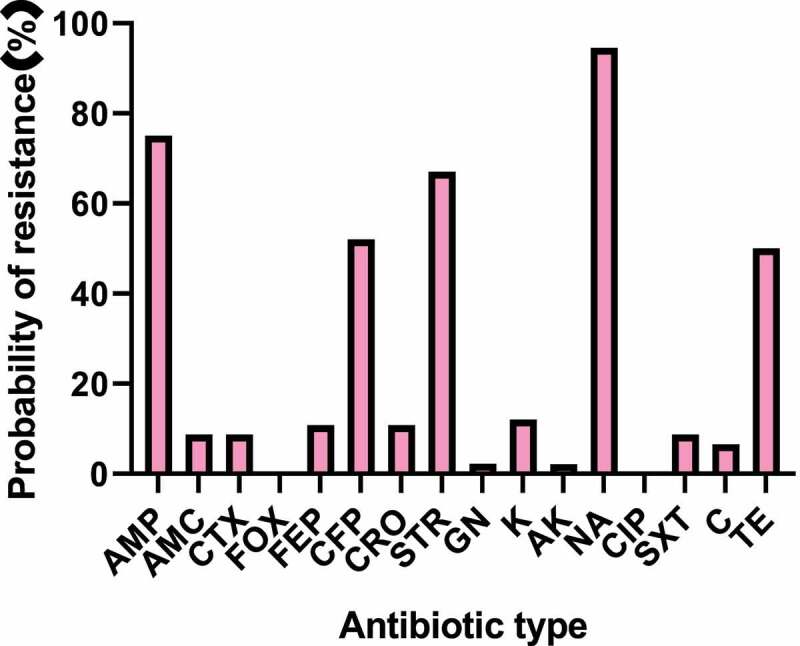
Drug resistance rates of *Salmonella enteritidis* to various antibiotics

**Figure 2.**
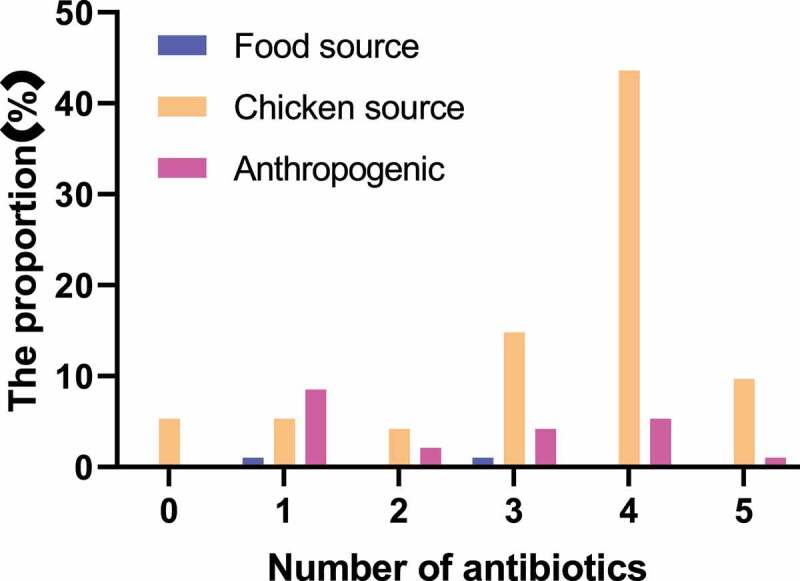
Statistical results of multi-drug resistance

The K-B disk result of *Salmonella enteritidis* strain was shown in Figures 1, 2 and Table 2. The main detected antibiotics were ampicillin (AMP), amoxicillin (AMC), cefoxitin (FOX), cefotaxime (CTX), cefoperazone (CFP), ceftriaxone (CRO), cefepime (FEP), streptomycin (STR), gentamicin (GN), kanamycin (K), amikacin (AK), nalidixic acid (NA), ciprofloxacin (CIP), sulfamethoxazole (SMZ), chloramphenicol (C, 30 μg), and tetracycline (TE). 97% of the strains were resistant to at least one antibiotic. The drug resistance rate of *Salmonella enteritidis* to nalidixic acid was the highest, followed by ampicillin, streptomycin, and cefoperazone, with the drug resistance rates of 94.5%, 75%, 67%, and 52%, respectively. Strains from different sources were not resistant to ciprofloxacin and cefoxitin. Except for the above antibiotics, the drug resistance rates of other drugs were about 2%-12%. The statistical results of drug resistance spectrum were shown in Figure 2. A total of 94 strains of *Salmonella enteritidis* formed 22 kinds of drug resistance spectrum. The strains were generally resistant to 4–5 kinds of antibiotics. Some strains mainly formed fixed drug resistance spectrum: AMP-CFP-STR-NA-TE (22.6, 21.7%), AMP-STR-NA-TE (17, 16%), AMP-CFP-STR-NA (11.1, 10.6%). The statistical results of multi-drug resistance were shown in Figure 3. The proportion of strains resistant to three or more kinds of antibiotics was 20.2%, 48.9% were resistant to four kinds of antibiotics, 14.9% were resistant to one kind of antibiotic, and 5.3% were resistant to five kinds of antibiotics.

**Table 2.** Test results of drug resistant bacteria spectrum

Fixed bacterial spectrum type	Number of resistant strains	Proportion (%)
None	5	5.38
NA	14	15.05
AMP-STR	1	1.08
STR-NA	3	3.23
STR-NA-TE	1	1.08
AMP-CFP-STR-NA	10	10.75
AMP-GN-K-NA	1	1.08
AMP-STR-NA-SXT	1	1.08
AMP-STR-NA-TE	16	16.13
AMP-NA-SXT-TE	1	1.08
AMC-FEP-CFP-STR-NA	1	1.08
AMP-AMC-CEP-STR-NA	2	2.15
AMP-AMC-CRO-NA-TE	1	1.08
AMP-CEP-STR-NA-SXT	1	1.08
AMP-CFP-STR-NA-TE	21	22.58
AMP-STR-NA-SXT-TE	1	1.08
AMP-AMC-K-NA-SXT-TE	1	1.08
AMP-AMC-CEP-STR-NA-TE	1	1.08
AMP-CFP-STR-NA-SXT-TE	3	3.23
AMP-CTX-FEP-CFP-CRO-K-NA	1	1.08
AMP-CTX-FEP-CFP-K-NA-C	6	6.45
AMP-AMC-FEP-CFP-CRO-STR-K-AK-NA-TE	1	1.08
AMP-AMC-CTX-FEP-CFP-CRO-GN-K-AK-NA	1	1.08

**Figure 3.**
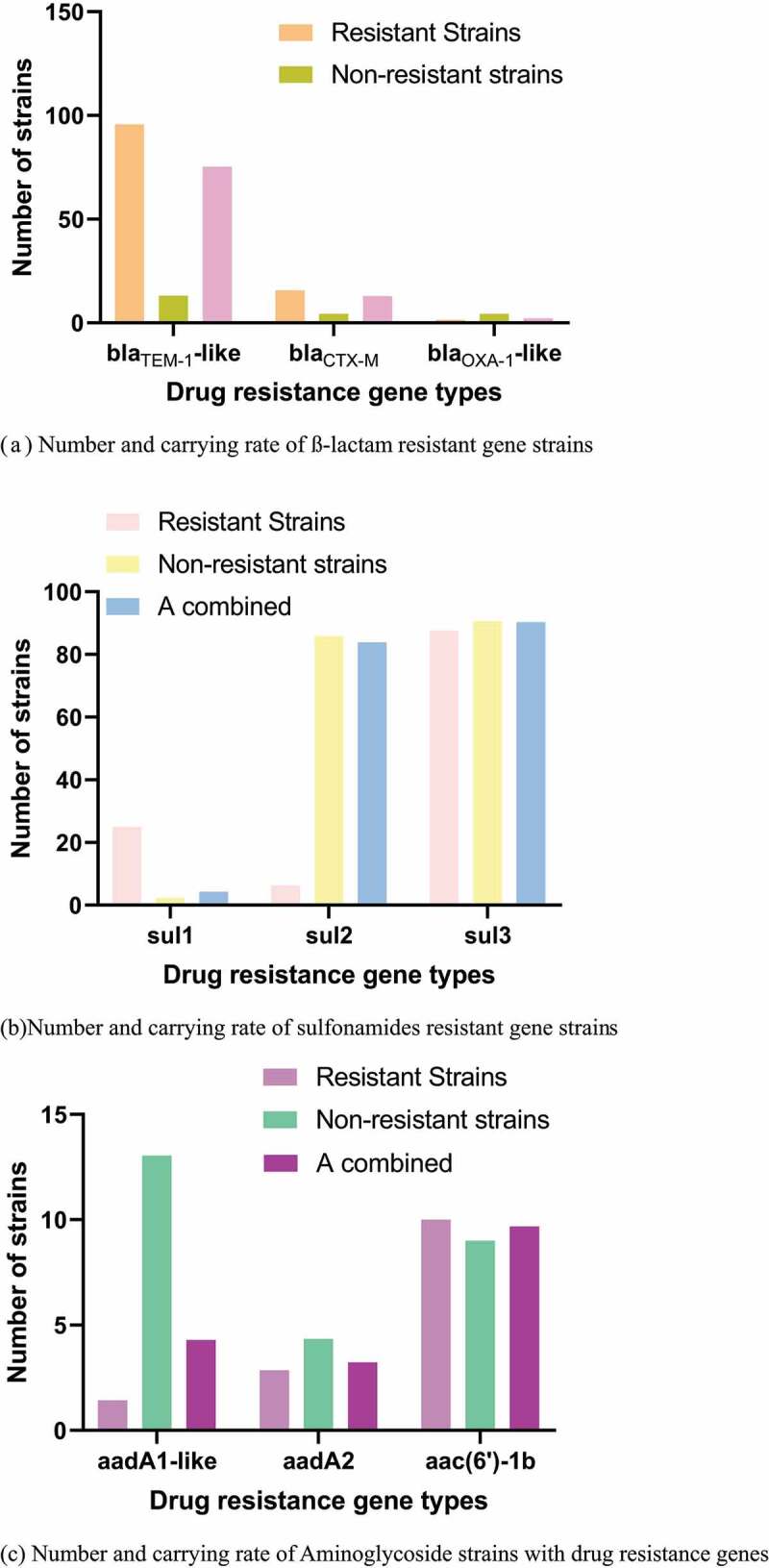
Number and carrying rate of strains of different antibiotics drug resistance genes. (a) Number and carrying rate of ß-lactam resistant gene strains. (b) Number and carrying rate of sulfonamides resistant gene strains. (c) Number and carrying rate of Aminoglycoside strains with drug resistance genes. (d) Number and carrying rate of trimethoprim resistant gene strains. (e) Number and carrying rate of strains of chloramphenicol resistant genes. (f) Number and carrying rate of tetracycline resistant gene strains. (g) Number and carrying rate of quinolone resistant gene strains

**Figure 3.**
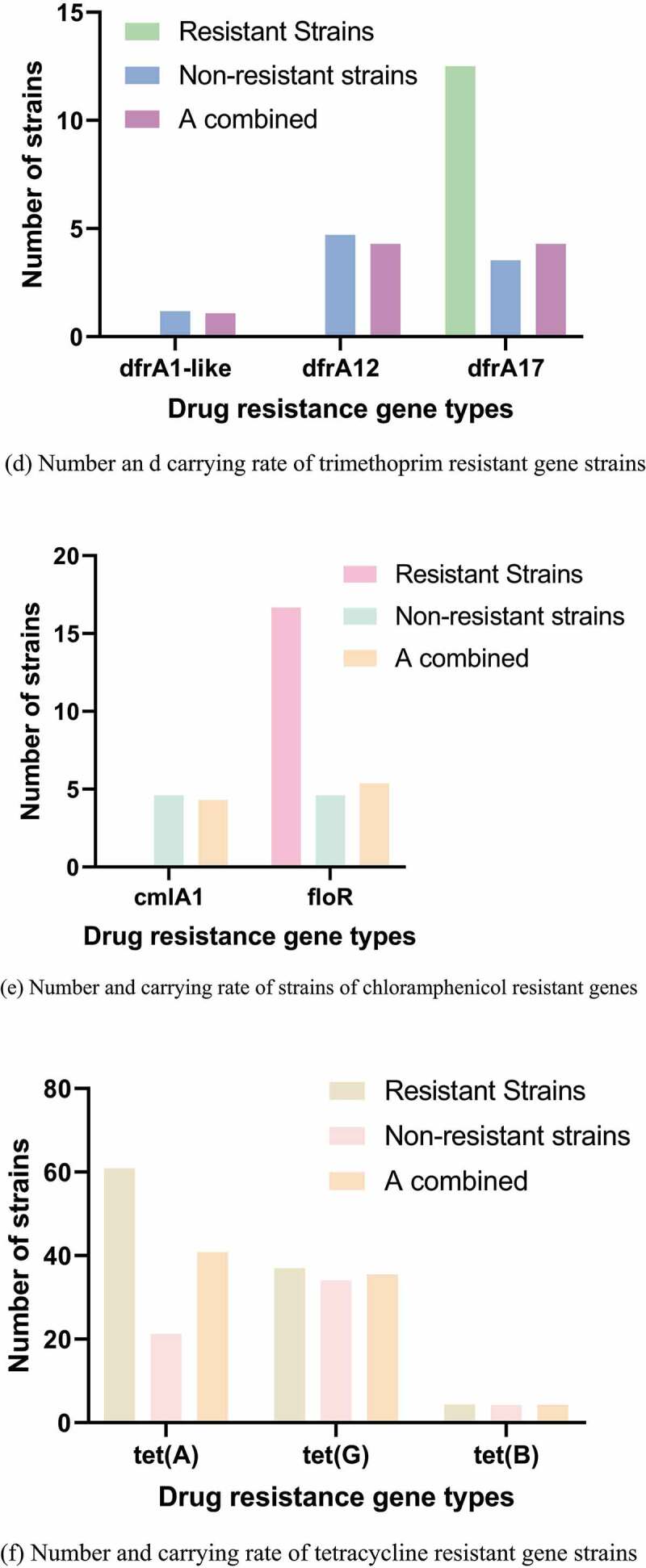
Continued

**Figure 3.**
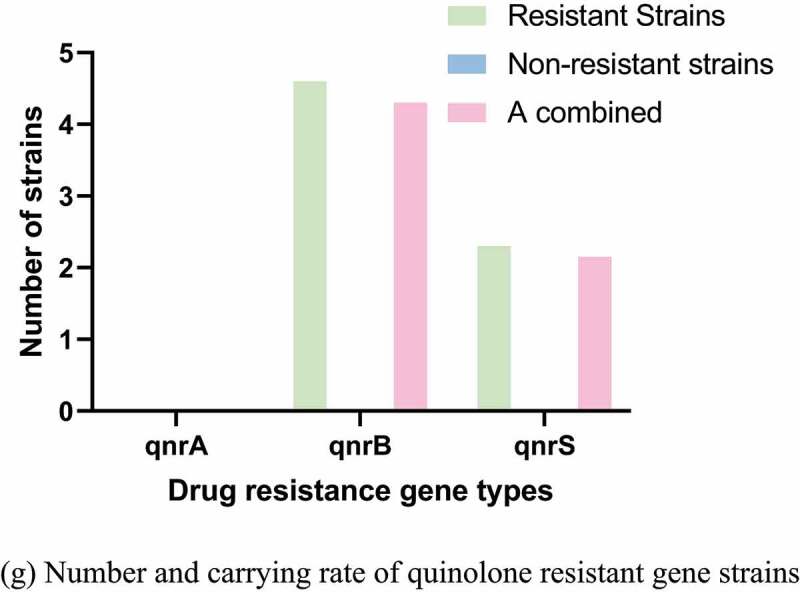
Continued

### 3.2. PCR detection results of drug resistance gene

The PCR detection results and distribution of drug resistance genes were shown in Figure 3. The carrying rates of blaTEM-1-like, blaCTX-M, and blaOXA-1-like drug resistance genes were 76%, 13.1%, and 2.16%, respectively. The carrying rates of sul1, sul2, and sul3 were 4.4%, 84.88%, and 90.33%, respectively. The carrying rates of aadA1-like, aadA2, and aac(6ʹ)-1b were 40.9%, 35.55%, and 4.33%, respectively. No tet(c) gene was detected. The carrying rates of cmlA1 and floR drug resistance genes were 4.3% and 5.38%, respectively, and no catA1 was detected.

### 3.3. Comparison of genes expression between biofilm group and planktonic bacteria group

**Figure 4.**
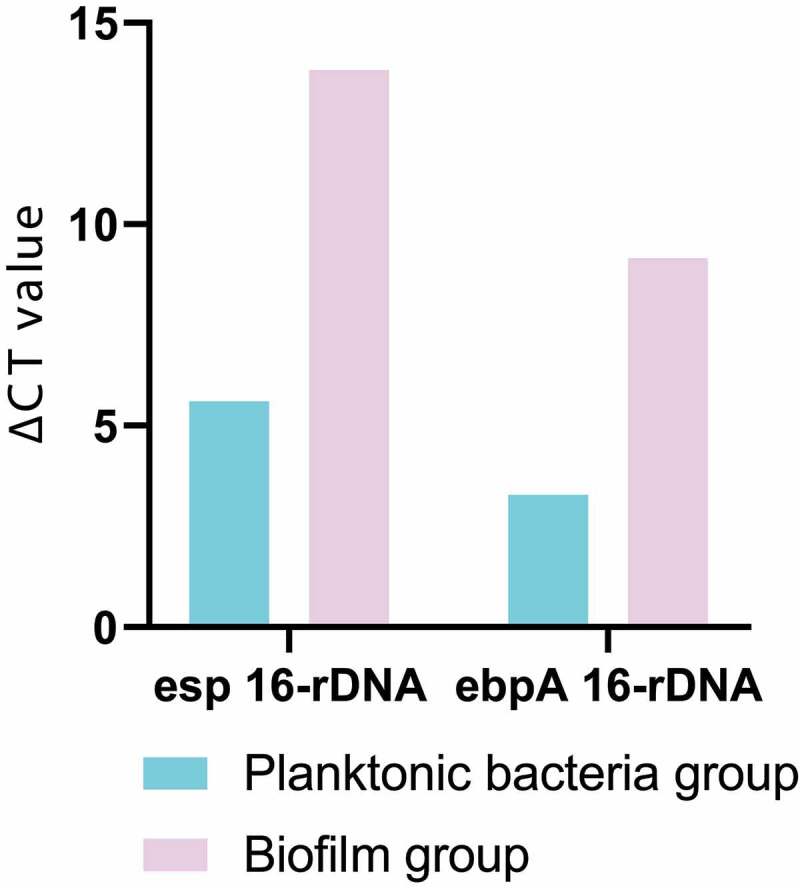
Analysis result of genes expression difference between the two groups

The ∆CT obtained by RT quantitative PCR was homogenized with 16s-rDNA, and then the target gene and 16s-rDNA were calculated according to the equation (amount of target = 2^−∆∆CT^), so as to further obtain ∆CT and complete data analysis. The results of data analysis were shown in Figure 4. The expression levels of enterococcal surface protein (esp) and enhancer binding protein-A (ebpA) in biofilm group were much higher than those in planktonic bacteria group, which were 299 times and 60 times higher than those in planktonic bacteria group, respectively.

### 3.4. Differences in gene expression of biofilm before and after induction and with the in vivo extraction

**Figure 5.**
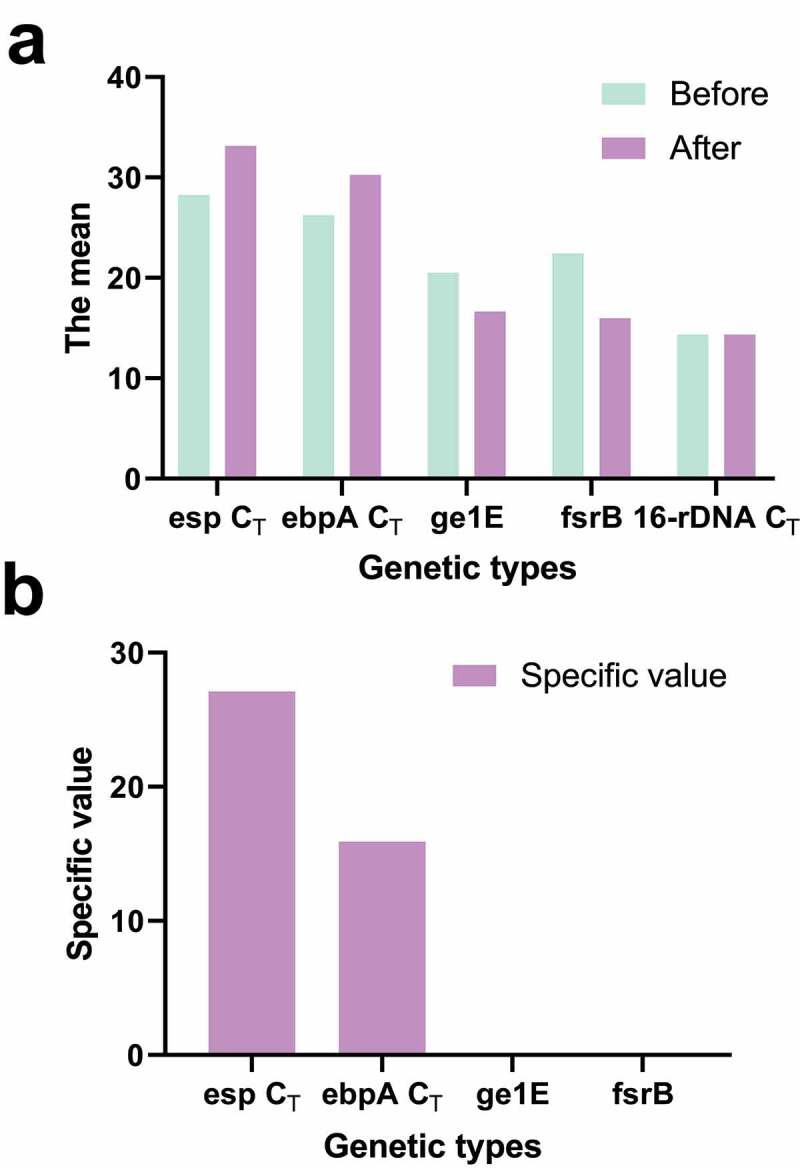
Genes expression changes of esp, ebpA, gl1E, and fsrB before and after induction

**Figure 6.**
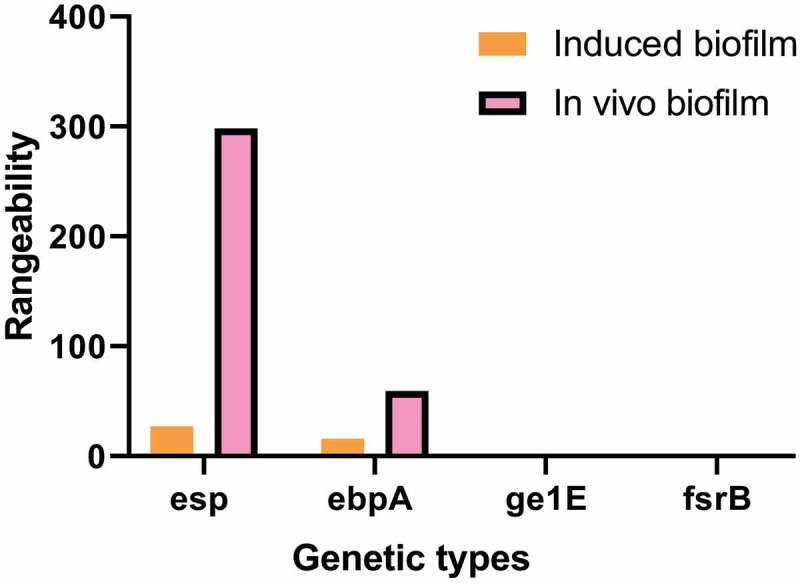
Comparison of gene expression changes of biofilm and induced biofilm in vivo

Figure 5 shows the difference in gene expression of the biofilm before and after the induction of the formation of the biofilm, and the difference in gene expression of the biofilm between the in vitro induction and the in vivo extraction of the biofilm gene. The expression levels of esp and ebpA were 28 times and 16 times those before induction. The expression levels of gl1E and fsrB were 1/15 and 1/89 of those before induction. The expression changes of esp, ebpA, gl1E, and fsrB before and after induction were significantly lower than those of biofilm formed in vivo (Figure 6).

(a): the average C_T_ value of each gene before and after induction; (b): expression ratio of genes before and after induction.

## 4. Discussion

At present, bacterial resistance has become a universally recognized problem. Multi-drug resistant bacterial pathogens, especially resistant to broad-spectrum cephalosporins and fluoroquinolones, have emerged and become prevalent worldwide. *Salmonella enteritis* is one of the pathogenic bacteria with serious drug resistance [15]. Clinical studies have shown that the strains are generally resistant to ampicillin, nalidixic acid, streptomycin, cefoperazone and tetracycline, which are currently widely used in clinical treatment [16]. In recent years, studies have been published on the serious health problems caused by multidrug-resistant *Salmonella* infections. For example, a large number of studies have shown that patients with *Salmonella* resistant to one or more clinically important antibiotics are three times more likely to have blood infections that require hospitalization compared with patients with common infections [17]. In addition, it was reported that patients with multidrug-resistant salmonella typhimurium infection were 4.8 times more likely to die than the general population, while patients with concurrent quinolone-resistant strains were 10 times more likely to die than the general population. *Salmonella enteritidis* is usually a less resistant serotype than *S. typhimurium*, and there were relatively few reports of multidrug-resistant *Salmonella enteritidis* previously, but with the increasing use of antibiotics in humans and animals, multidrug-resistant *Salmonella enteritidis* has emerged. With the increasing use of antibiotics, in addition to the increasing resistance of bacteria, drug-resistant genes also arise [18]. In this paper, *Salmonella enteritis* was selected as the research object, and its tolerance and the carrying situation of drug-resistant genes were analyzed. The results showed that the drug resistance rates of *Salmonella* enteritis to nalidixic acid, ampicillin, Streptomyces and cefoperazone were 94.5%, 75%, 67%, and 52%, respectively. A total of 94 strains of *Salmonella enteritidis* formed 22 kinds of drug resistance spectra, the strains were generally resistant to 4–5 antibiotics, and some strains mainly formed fixed drug resistance spectra: Amp-cfp-str-na-te (22.6, 21.7%), AMP-STR-NA-TE (17, 16%), and AMP-CFP-STR-NA (11.1, 10.6%), and they were consistent with previous studies. The results showed that sul2, sul3, blaTEM-1-like, tet(A), tet(G) genes were common drug-resistant genes in *Salmonella enteritis*.

Bacterial biofilm (BBF) is a unique life phenomenon conducive to the survival of bacteria in order to adapt to the natural environment. The study found that most natural bacteria can exist in the form of biofilm, and *Salmonella enteritis* can also form biofilm. The production of biofilm makes the bacteria in the membrane not only more resistant to antibiotics than plankton 10–1000 times, but also resistant to the bactericidal action of antibodies. Biofilms are the main cause of some refractory infections. Biofilm is a colony organization composed of many microcolonies, and there is a strict information transmission system among the tissues. This system is called bacterial quorum sensing. In recent years, the research on bacterial quorum sensing system has been deepening, and a large number of research results show that it plays an important role in regulating the formation, development and function of biofilms [19]. As one of the common pathogenic bacteria in clinic, there are many researches on the quorum sensing system of *Salmonella enteritis* biofilm bacteria. Some foreign scholars have shown that the quorum-sensing system of *Salmonella enteritis* is its virulence regulator QS-FSR system. However, it is not clear which genes are involved in the formation of *Salmonella enteritis* biofilm and how qS-FSR system regulates biofilm formation through these genes [20]. In this paper, we induced the formation of biofilms in the laboratory and compared their gene expression with that of related organisms extracted in vivo. The results showed that sp, ebpA, ge1E and fsrB genes were closely related to the formation of *Salmonella enteritidis* biofilm. Qs-fsr system could regulate the expression of ESP, ebpA and gelE genes, reduce the expression of ESP, ebpA and gelE, and increase the expression of gelE.

## 5. Conclusion

In this research, *Salmonella enteritidis* was taken as the research object, and its resistance to antibiotics and biofilm formation mechanism were studied. It was found that the resistance rate of *Salmonella enteritidis* to nalidixic acid, ampicillin, streptomyces, and cefoperazone was high. esp, ebpA, ge1E, and fsrB genes were closely related to the biofilm formation of *Salmonella enteritidis*. In addition, the formation of biofilm can be controlled by regulating esp, ebpA, ge1E, and fsrB.

## 6. Deficiency and prospect

Due to the limitations of experimental conditions and personal knowledge, there are still many deficiencies in this study. For example, the experimental results of this study show that there are differences in gene expression between the biofilm extracted in vitro and the biofilm induced in the laboratory. This shows that the simulated induction in vitro cannot completely restore the environment of biofilm formation in vivo. In the future study and work, the experimental conditions will be further optimized in order to highly restore the biofilm formation process in vivo, so as to better explain the mechanism of biofilm formation.
